# A Study to Determine the Incidence and Distribution Patterns of Foot and Ankle Tumors in Bone and Soft Tissue

**DOI:** 10.7759/cureus.25598

**Published:** 2022-06-02

**Authors:** Selami Karadeniz, Alparslan Yurtbay, Bedirhan Albayrak, İsmail Büyükceran, Nevzat Dabak

**Affiliations:** 1 Orthopedics and Traumatology, Amasya University, Amasya, TUR; 2 Orthopedics and Traumatology, Samsun Education and Research Hospital, Samsun, TUR; 3 Orthopedics and Traumatology, Ondokuz Mayis University, Samsun, TUR

**Keywords:** diagnosis, bone neoplasms, soft tissue neoplasms, ankle joint, foot

## Abstract

Objectives: The aim of this study is to determine the incidence and distribution patterns of foot and ankle tumors in bone and soft tissue in a university tumor institute, to help the correct evaluation of uncertain masses, to take the right steps in advanced diagnosis and treatment, and to contribute to future research.

Materials and methods: A retrospective analysis of a total of 164 foot and ankle cases examined by a multidisciplinary bone and soft-tissue tumors care team between January 2004 and December 2021 was performed from a database in which patient information was recorded in our tertiary university hospital. Thirty-three (20.1%) of 164 patients were discussed in the tumor council and evaluated as having the non-tumor disease. All of these patients were excluded from the study. A total of 131 patients diagnosed with tumors were included in this study.

Results: The lesion was determined as a benign tumor in 84 (64.1%) cases of 131 tumor patients included in the study. Of these 84 patients, 40 (47.6%) were identified as benign bone lesions and 44 (52.4%) as benign soft-tissue lesions. Malignancy was determined in 47 (35.9%) of 131 patients, affecting the bone in 14 (29.8%) patients and the soft tissue in 33 (70.2%). The malignant soft-tissue lesion most determined was malignant mesenchymal tumor in 10 (30.3%) patients, of which one had lung metastasis and one was determined with multiple metastases. Metastasis was detected in eight patients in total, including three metastatic malignant bone tumors and five metastatic malignant soft-tissue tumors.

Conclusions: Tumors involving the foot and ankle are not frequently encountered, and most tumors in this region are benign. The anatomic structure of the foot allows early diagnosis, but for diagnosis to be made, there must first be clinical suspicion. The first symptom is generally swelling. Early diagnosis can prevent several complications. Therefore, patients with foot and ankle complaints must be taken seriously and evaluated with advanced tests if necessary.

## Introduction

Bone and soft-tissue tumors of the musculoskeletal system are rarely seen when the most common neoplasms such as breast, colon, and lung cancer are compared. Tumors and tumor-like lesions (pseudotumors) are seldom encountered in the foot and ankle. Previous studies have reported that 5-10% of all musculoskeletal tumors are determined in the foot and ankle [1-4]. Considering the ratio of tissue volume to whole-body volume, it is remarkable that there is an unexpectedly high prevalence. Malignant tumors and metastases around the foot and ankle are rarely seen. The majority of lesions in this region originate from soft tissue and are benign [5]. Underestimating the malignant potential of a tumor located in this region, the diagnosis of many malignant tumors will be delayed despite early symptom presentation in the disease process.

The complaints of patients are generally non-specific, and the most common reasons for presentation are pain and swelling. Tumors can be noticed in the early stage due to the compact anatomy of the ankle, and tumors in this region can be easily palpated. Although the ankle anatomy provides an advantage for the early diagnosis of tumors, as these tumors are rarely seen, clinical awareness is not sufficient. It can be overlooked that a lesion seen in the foot or ankle can have the malignant potential [3,6]. Errors in diagnosis are more common than in other parts of the body because it is often not thought to be a true neoplasia. Neoplasia should be considered as a differential diagnosis in any unclear, persistent swelling or bone lesion to ensure correct and early diagnosis and appropriate treatment. The surgeon, who deals with the foot and ankle region, evaluates the relevant differential diagnoses and is responsible for initiating the necessary steps for further diagnosis and treatment.

Inadequate and incomplete treatment has a negative impact on oncological and functional treatment outcomes [7]. In addition, in patients planning to undergo surgery, the ankle anatomy does not allow wide resection to be made at the desired rate. These tumors can sometimes result in metastasis and extremity amputation.

The aim of our study was to report the results of a retrospective, epidemiological study of bone and soft-tissue tumors located around the foot and ankle in patients treated at the Ondokuz Mayis University Department of Orthopedics and Traumatology (Samsun, Turkey). The primary aim of this study can be listed as to determine the incidence and distribution patterns of foot and ankle tumors in bone and soft tissue in a university tumor institute, to help the correct evaluation of uncertain masses, to take the right steps in advanced diagnosis and treatment, and to contribute to future research.

## Materials and methods

Following Institutional Ethics Committee approval, a retrospective analysis of a database retained prospectively at our tertiary university hospital was performed. Our institutional archive includes original records, including clinical documentation and imaging studies, for patients receiving treatment or consultation at the Ondokuz Mayis University Department of Orthopedics and Traumatology (Samsun, Turkey) since 2004. Histopathologically confirmed diagnoses and tissue samples are available by the pathology department.

Five thousand one hundred and two cases were recorded from the database start (January 2004) to December 2021. For this research, we reviewed the database to identify all patients with foot or ankle tumors treated from January 2004 to December 2021. Primary lesions involving the foot and/or ankle, a histologically confirmed diagnosis, and patients who underwent biopsy and treatment (when performed) in our institution were included in the study. Patients were excluded from the study if medical record data, imaging studies, or histological slides were missing, resulting in an unclear or inadequate description of a tumor. Informed consent is obtained from all patients at the time of admission to our institution to be included in scientific studies. Ondokuz Mayis University Institutional Review Board approved (decision no: OMU KAEK: 2022/128, dated: 18.04.2022) the protocol for our research.

A retrospective analysis of a total of 164 foot and ankle cases examined by a multidisciplinary bone and soft-tissue tumors care team between January 2004 and December 2021 was performed from a database in which patient information was recorded in our tertiary university hospital. Thirty-three (20.1%) of 164 patients were discussed in the tumor council and evaluated as having the non-tumor disease. All of these patients were excluded from the study. A total of 131 patients diagnosed with tumors were included in this study.

The patients with pain or swelling in the foot and ankle who were discussed in the multidisciplinary bone and soft-tissue tumors care team were examined in the study. Lesions showing localization from the toes to 10 cm proximal of the tibiotalar joint were evaluated as foot and ankle lesions. Information of the patients on the bone and soft-tissue tumor council form of age, gender, complaint, pre-diagnosis, definitive diagnosis, and bone and soft-tissue tumor council decision was recorded.

The clinical findings, radiological images if available, and pathological results of the patients were evaluated. The necessary tests and treatment to be applied were decided in accordance with the opinions of the multidisciplinary bone and soft-tissue tumors care team. The bone or soft-tissue tumors of all the patients in the study were diagnosed radiologically and/or pathologically. The patients were grouped as benign soft-tissue tumors, benign bone tumors, malignant soft-tissue tumors, malignant bone tumors, metastatic tumors, and diseases other than the tumor.

Data obtained in the study were analyzed statistically using SPSS for Windows version 21.0 software (SPSS ınc., Chicago, IL, USA). Statistical values were stated as mean ± standard deviation values, number (n), and percentage (%). Categorical variables are expressed as the frequency count and percentage of the total number of lesions in a specified category.

## Results

An evaluation was made of 164 patients comprising 81 males and 83 females with a mean age of 39.1±22.13 years (range, 3-82 years). Thirty-three (20.1%) of 164 patients were discussed in the tumor council and evaluated as having a non-tumor disease. All of these patients were excluded from the study. A total of 131 patients diagnosed with tumors were included in this study. 

In the remaining 131 patients, the lesions were determined as a benign tumor in 84 (64.1%) cases, of which 40 were benign bone lesions and 44 were benign soft-tissue lesions. The benign bone lesions comprised six osteochondroma, six aneurysmal bone cysts, five osteoid osteoma, four intraosseous hemangioma, four non-ossified fibroma, four solitary bone cysts, and others (Table 1).

**Table 1:** Distribution of benign bone tumor lesions of the foot and ankle (n: 40 lesions in 131 patients)

Benign Bone Tumors	Number of Patients (n=40)	%
Osteochondroma	6	15
Aneurysmal Bone Cyst	6	15
Osteoid Osteoma	5	12.5
Intraosseous Hemangioma	4	10
Non-ossified Fibroma	4	10
Solitary Bone Cyst	4	10
Intraosseous Lipoma	2	5
Chondroblastoma	2	5
Monostatic Fibrous Dysplasia	1	2.5
Hamartoma	1	2.5
Enchondroma	1	2.5
Giant Cell Tumor	1	2.5
Intraosseous Mixoma	1	2.5
Adamantinoma	1	2.5
Osteoblastoma	1	2.5

Treatment for the patients was planned according to age, tumor localization, and clinical status. Cystic lesions with no clinical complaints and that did not create cortical destruction or constitute a risk for fracture were followed up conservatively. In the cases requiring surgery, the tumor was totally removed with curettage and grafting applied. Amputation was performed in two patients diagnosed with adamantinoma and giant cell bone tumor.

Of the 44 patients with benign soft-tissue tumor, ganglion cyst was determined in 10, lipomatous changes in seven, plantar fibromatosis in seven, hemangioma in six, giant cell tumor of the tendon sheath in five, schwannoma in three, synovial chondromatosis in two, and parosteal lipoma, myositis ossificans, leiomyoma, and lymphangioma in one patient each (Table 2). Conservative follow-up was recommended for five patients with myositis ossificans, lymphangioma, and hemangioma. Synovectomy was applied by performing arthrotomy in two patients with synovial chondromatosis. In the remaining 37 patients, the tumor was excised.

**Table 2:** Distribution of benign soft-tissue tumor lesions of the foot and ankle (n: 44 lesions in 131 patients)

Benign Soft-Tissue Tumors	Number of Patients (n=44)	%
Ganglion Cyst	10	22.7
Lipomatous Changes	7	15.9
Plantar Fibromatosis	7	15.9
Hemangioma	6	13.6
Tendon Sheath Giant Cell Tumor	5	11.3
Schwannoma	3	6.8
Synovial Chondromatosis	2	4.5
Parosteal Lipoma	1	2.2
Myositis Ossificans	1	2.2
Leiomyoma	1	2.2
Lymphangioma	1	2.2

Malignancy was determined in 47 (35.9%) of 131 patients, affecting the bone in 14 patients and the soft tissue in 33. The malignant bone tumor most determined was Ewing's sarcoma in 10 patients, of which two had lung metastasis and one had metastasis in the shoulder and acetabulum. Chondrosarcoma was determined in two patients and osteosarcoma in two, and amputation at an appropriate level was performed in these patients (Table 3). Metastasis was detected in eight patients in total, including three metastatic malignant bone tumors and five metastatic malignant soft-tissue tumors.

**Table 3:** Distribution of malignant bone tumor lesions of the foot and ankle (n: 14 lesions in 131 patients)

Malignant Bone Tumors	Number of Patients (n=14)	%
Ewing's Sarcoma	10	71.4
Osteosarcoma	2	14.2
Chondrosarcoma	2	14.2

The malignant soft-tissue lesion most determined was a malignant mesenchymal tumor in 10 patients, of which one had lung metastasis and one was determined with multiple metastases. In five patients, the mass was in the plantar region, and in the other three, in the toes. Treatment was applied with appropriate level amputation in five patients and wide resection in three. A diagnosis of malignant melanoma was made in nine patients, of which three were acral melanoma (Table 4). 

**Table 4:** Distribution of malignant soft-tissue tumor lesions of the foot and ankle (n: 33 lesions in 131 patients)

Malignant Soft-Tissue Tumors	Number of Patients (n=33)	%
Malignant Mesenchymal Tumor	10	30.3
Malignant Melanoma	9	27.2
Synovial Sarcoma	6	18.1
Squamous Cell Carcinoma	4	12.1
Fibrosarcoma	2	6
Kaposi Sarcoma	1	3
Pleomorphic Undifferentiated Sarcoma	1	3

Metastasis was determined in eight patients. In four of these patients, the primary tumor was in the lungs. Metastasis was determined from rectum cancer in one patient, from renal cell cancer in one, and from B cell lymphoma in two.

## Discussion

Tumors and tumor-like lesions (pseudotumors) are seldom encountered in the foot and ankle. The frequency of foot and ankle tumors has been reported in the range of 5-10% in the literature [1-4]. Few large series have been published as these tumors are uncommon. A case series of 39,179 patients reported that 8% of benign soft-tissue tumors and 5% of malignant soft-tissue tumors were localized in the foot and ankle [8]. In another series of 1452 patients, foot and ankle tumor localization was reported in 75 (5.1%) patients [9]. There are few studies on this subject in the literature [3,9-13]. We think that this study will make an important contribution to the literature.

Early diagnosis and treatment are known to have a positive effect on clinical outcomes, especially in high-grade malignancies. The anatomic structure of the ankle allows early diagnosis of tumors. As the muscle layers are not thick and the skin structure and the subcutaneous fat tissue are thin, these tumors can be easily palpated and may become symptomatic in very early stages. Pain starts in the early period. However, since tumors in this region are rarely seen, clinicians' awareness may not be sufficient. In literature, problems have been reported associated with delayed diagnosis and incorrect treatment [14,15]. This and similar studies will help to increase the awareness of clinicians. 

Tumors involving the foot and ankle are usually benign and originate from soft tissue. Soft-tissue ganglion cysts are most common, followed by plantar fibromatosis [16]. In this study, similar to the literature, the most common benign soft-tissue tumor was a ganglion cyst, followed by lipomatous changes and plantar fibromatosis.

Soft-tissue sarcomas usually begin as a small, asymptomatic swelling and are often underestimated. The most important stage of diagnosis is clinical suspicion. Any swelling detected should be noted and necessary tests should be performed. Foot and ankle involvement has been reported in 4% of malignant soft-tissue tumors, of which 45% are synovial sarcoma [17]. Synovial sarcoma was found to be the third most common malignant soft-tissue tumor (18.1%) in the current study. Treatment of soft-tissue sarcomas depends on the tumor grade. Enneking grade 1 and grade 2 lesions can be treated with en bloc marginal excision, and grade 3 and 4 lesions usually require amputation [18,19]. In this study, an appropriate level of amputation was performed for stage 3 and 4 grade lesions with malignant masses on the toes. En bloc resection was performed in two patients with Enneking stage 1 malignant mesenchymal tumors.

In literature, osteochondroma has been reported to be the most frequently seen primary benign bone tumor [17]. However, in a study by Murari et al. of 297 patients, the rate of osteochondroma was found to be 6%, and the most frequently encountered primary benign bone tumor at the rate of 19.2% was reported to be chondromyxoid fibroma [20]. The most common primary benign bone tumors in this study were osteochondroma (15%) and aneurysmal bone cyst (15%).

Malignant bone tumors of the foot and ankle are rare, and the most common malignant bone lesions of the foot and ankle are metastatic lesions, Ewing's sarcoma, and chondrosarcoma [10,21,22]. In a previous study, chondrosarcoma was reported to be the most common primary malignant bone tumor of the foot, detected in 22 (52.3%) of 42 patients [20]. In the current study, Ewing's sarcoma was found to be the most common malignant bone tumor. Of the 14 malignant bone tumors identified in the current study, Chopart amputation was performed in one, toe amputation in two, Syme amputation in two, and tibia amputation in four patients. Because of the foot and ankle anatomy, it is very difficult to perform limb-sparing surgery in the treatment of malignant bone tumors. Although wide resection can be applied to tumors detected in Enneking stage 1, the gold standard treatment for foot and ankle tumors is amputation at the right level.

Metastatic lesions are the most commonly seen malignant bone lesions in the musculoskeletal system. However, foot and ankle metastasis is rare, although lesions may be seen in every region of the foot. In a study of 41,833 cancer patients, metastasis in the foot and ankle was only seen in four patients [23]. In another study by Hattrup et al., 75,000 cancer patients were examined, and metastasis in the foot and ankle was determined in 10 patients [24]. A total of 204 patients with foot and ankle tumors were examined in another study and a malignant lesion was determined in 41, and six of these were reported to be metastatic lesions [10]. In the current study, malignancy was determined in 47 (35.9%) of 131 patients. Of these, eight were the result of metastasis, four of which were determined to be from the lungs (three adenocarcinoma, one epidermoid carcinoma). It can be seen from the literature that the tumors with the most frequent metastasis in the foot and ankle originate from the lungs [25]. Hattrup et al. reported that the tumors metastasizing most frequently in the foot and ankle were of the lung, kidney, and gastrointestinal region origin [24]. Consistent with these findings in the literature, lung-origin metastases were seen most often in the current study, and metastasis from rectum carcinoma in one patient and from renal cell carcinoma in one. In addition, metastasis from B-cell lymphoma was determined in two patients. The rates of metastasis in the foot and ankle in the current study were found to be higher than the rates reported in the literature.

There were some limitations to this study, primarily the retrospective design and that foot and ankle tumors are not common, so the sample number was low. A number of methodological shortcomings in diagnosing a tumor, documenting the specific characteristics of the tumor, and interpreting the documented data may have affected our results. Almost all of the patients were treated by the researchers.

## Conclusions

In conclusion, tumors involving the foot and ankle are not frequently encountered, and most tumors in this region are benign. The anatomic structure of the foot allows early diagnosis, but for diagnosis to be made, there must first be clinical suspicion. The first symptom is generally swelling. Early diagnosis can prevent several complications. Therefore, patients with foot and ankle complaints must be taken seriously and evaluated with advanced tests if necessary.
